# Interest in and Predictors of Engagement With a Virtual Reality Intervention Among People With Chronic Pain: Cross-Sectional Survey Study

**DOI:** 10.2196/70598

**Published:** 2026-02-11

**Authors:** Genevieve R Bryant, Samuel A Holzman, Hector R Perez

**Affiliations:** 1 SUNY Downstate College of Medicine Brooklyn, NY United States; 2 Albert Einstein College of Medicine Bronx, NY United States

**Keywords:** chronic pain, virtual reality, barriers to treatment, technology-based intervention, accessibility

## Abstract

**Background:**

Although chronic pain (CP) is highly prevalent, current modalities are not sufficient to address the needs of people living with this condition. Pharmacological treatments for CP can have severe side effects and increased likelihood of patients overdosing or developing addiction. Behavioral treatments are often indicated for the treatment of CP, but barriers to treatment are common. Virtual reality (VR)–based interventions have shown promise as an effective and potentially accessible form of treatment for CP. However, previous research on VR interventions for people living with CP has not often included diverse populations, including racial and ethnic minority groups and people with low socioeconomic status.

**Objective:**

This study aimed to gauge the interest of patients with CP in participating in a hypothetical study of at-home VR for CP and to identify predictors of interest. Patients were recruited from a low socioeconomic and racially and ethnically diverse community.

**Methods:**

A total of 48 participants living with CP were recruited from an electronic medical record database, a research participant database, and a pain clinic, and they completed surveys about demographics, pain levels, technology use, and knowledge of VR. Bivariate testing was used to determine which, if any, of the aforementioned variables were associated with interest in a hypothetical study of at-home VR for CP. Stepwise logistic regression models predicting interest were built based on bivariate testing. Finally, we used a thematic analysis framework to analyze an additional open-ended question about reasons for interest in participating in a VR intervention for CP.

**Results:**

Despite low technology use and little knowledge and experience with VR, results showed high interest (42/48, 88%) among patients in participating in a hypothetical study of at-home VR for CP. More frequent email use and using Facebook demonstrated nonsignificant trends toward interest in participating in a VR clinical trial for pain (*P*=.06 for email use and *P*=.06 for Facebook use). In stepwise multivariate models controlling for pain score, Facebook use was predictive of being somewhat or very interested in participating in a VR clinical trial for pain (*P*=.047). Open-ended responses tended to cite the novelty of VR and desperation for pain relief as reasons for participants’ interest.

**Conclusions:**

We found high interest in participating in a clinical trial of VR despite low use of technology and low knowledge of VR. Future fully powered studies should seek to confirm the effectiveness of VR treatments for people with CP, especially people from lower socioeconomic, and racially and ethnically diverse backgrounds.

## Introduction

Chronic pain (CP) affects an estimated 50.2 million adults in the United States [1], and it remains one of the most common reasons people seek medical care [2]. Individuals who live in poverty, have less than a high school education, and are on public health insurance are more likely to have high-impact CP [3]. Moreover, CP is linked to numerous adverse physical and mental health outcomes [3].

Despite the burden of CP, there is no gold standard treatment, and current options are insufficient for many people living with CP. Many medications, such as antidepressants and antiepileptic drugs, have severe side effects [4]. Patients are often dissatisfied with conventional pharmacological pain treatment, reporting that CP continues to affect their health, relationships, happiness, and quality of life [5]. Opioid medications can be ineffective and carry significant risk for patients, including the potential for addiction and overdose [6,7]. Nonpharmacologic options are recommended for treating CP, but barriers to accessing them are common, including lack of availability, distance to travel, and high cost of treatment [8].

In numerous studies, patient participants seek new, innovative treatment options for CP. Virtual reality (VR) is a promising new category of therapeutic options for pain management without many of the drawbacks of conventional CP treatments. VR is effective in the treatment of acute pain [9], and emerging evidence suggests it can be effective in the treatment of CP [10]. Additionally, portable VR headsets can be used at home or in a clinic, potentially increasing availability and accessibility of treatment. Interventions involving VR may also become more affordable and more widely available compared with other current treatments as the cost of such technology continues to decrease [11,12].

As VR technology develops, the need for robust study is paramount. Nonetheless, racial and ethnic minority groups and individuals of lower socioeconomic status (SES) may face barriers to entering new VR clinical trials due to preexisting disparities in pain clinical trial enrollment and, potentially, due to low interest in technology interventions [13]. Most VR clinical trials for individuals with CP in the United States to date have included samples primarily composed of non-Hispanic White, high-SES participants [14,15].

To ensure effectiveness in all populations and to ensure equity, further clinical studies of diverse patient populations are necessary. As part of preparations for recruiting participants to a pilot study of VR for CP, we conducted a cross-sectional study of patients with CP in a low SES and racially and ethnically diverse county. The survey was aimed at identifying patients’ interest in participating in a hypothetical at-home VR trial and factors associated with interest.

## Methods

### Overview

We conducted a cross-sectional study using telephone and in-person surveys among patients with CP to evaluate interest in participating in a study of VR for pain management and to examine factors associated with interest.

### Ethical Considerations

The study was approved by the Albert Einstein College of Medicine and Montefiore Medical Center Institutional Review Board through the Office of Human Research Affairs (2021-13108**)**. Oral informed consent was obtained over the telephone at the time of the interview. Identifying data was omitted if not essential and are not published in this manuscript. Participants were reimbursed US $10 in electronic gift cards for their time at the conclusion of the survey.

### Participants

We recruited patients with CP in the Bronx, New York, both over the phone and in person. Bronx County is the poorest county in New York State and has the lowest internet adoption rates of any borough in New York City [16]. One-third of the Bronx population is Black or African American, 55% is Hispanic or Latino, and 9% is non-Hispanic White [17]. The median household income is US $45,517, with 27.7% of the Bronx population living in poverty [17].

### Recruitment and Eligibility

There were 3 methods used for recruitment. First, we used hospital databases to identify potential participants based on *International Classification of Diseases, Tenth Revision (ICD-10)* codes. Second, we used a separate database that consisted of patients with CP that had completed previous research studies at our institution and consented to be recontacted for future research opportunities. Potential participants were identified from these 2 databases and contacted over the phone. Third, patients were recruited in person from a general medicine CP clinic. To be eligible to complete the survey, patients had to be aged at least 18 years, speak English fluently, and be able to provide consent for themselves.

### Data Collection

Data were collected between August 2021 and December 2022 and deidentified for analysis. Participants completed the following questionnaires.

#### Sociodemographic Questions

We asked participants about their age, highest educational level, and race and ethnicity. Educational level was dichotomized into 2 groups: less than high school education and high school education or higher.

#### Pain Questions

We asked participants about their CP using the Pain, Enjoyment of Life, and General Activity (PEG) scale [18]. The PEG scale is a brief 3-item scale that assesses pain intensity and interference. The 3 questions assess pain on average in the past week, how much pain has interfered with their general activity, and how much pain has interfered with their enjoyment of life. Answer choices for each of the 3 questions were given on a 0- to 10-point scale, where 0 was “no pain” or “it does not interfere” and 10 was “pain as bad as you can imagine” or “it completely interferes.” The PEG score is calculated using the mean of the answers to the 3 questions. Additionally, we asked participants about the level of bodily pain they had experienced in the past week. The answer choices were “none,” “very mild,” “mild,” “moderate,” “severe,” and “very severe.” We also asked participants about all locations where they experienced pain for at least the last 3 months.

#### Media and Technology Usage Scale

We asked participants about their technology use and attitudes about technology using the Media and Technology Usage Scale [19]. This scale measures (1) the use frequency of a variety of technology and media types and (2) attitudes toward technology. There are 11 usage subscales representing smartphone use, general social media use, internet searching, emailing, media sharing, SMS text messaging, video gaming, online friendships, Facebook (Meta Platforms) friendships, phone calling, and watching television. Answers to questions related to these 11 subscales are scored on a 10-point frequency scale: 1=never, 2=once a month, 3=several times a month, 4=once a week, 5=several times a week, 6=once a day, 7=several times a day, 8=once an hour, 9=several times an hour, 10=all the time. Notably, the smartphone usage scale described above reflects using functions of the smartphone, such as apps, and does not reflect using the smartphone for calling or texting; calling and texting are represented in separate scales. Attitudes toward technology are rated on a 5-point Likert-type scale ranging from 1 (strongly disagree) to 5 (strongly agree). Questions in this domain include (1) positive attitudes toward technology, (2) negative attitudes toward technology, and (3) anxiety or dependence on technology.

#### VR Questions

In total, we asked 6 questions about VR. First, we asked participants a set of four questions about their experience and perception of VR: (1) whether they had ever heard of VR, (2) whether they had used a VR device, (3) whether they had heard of VR being used to treat pain, and (4) whether they thought VR could work for treating pain. Participants could answer yes or no for the first 3 questions and had the additional option of answering not sure for the fourth question. A fifth question asked if participants would be interested in participating in a hypothetical study of at-home VR for CP and could answer that they were “not interested,” “somewhat interested,” or “very interested.” A sixth question was an open-ended question for participants to provide reasons for their interest or lack of interest.

### Data Analysis

All participant data were included in the analysis. Information about gender was extracted from the electronic medical record. We calculated descriptive statistics (mean, median, and SD) for the PEG score, sociodemographic measures, and each of the subscales on the Media and Technology Usage Scale.

Our dependent variable was interest in participating in a hypothetical at-home VR study for CP. For this outcome, we dichotomized responses as “not interested” vs “somewhat interested” or “very interested.” While our dependent variable is subjective, in this open-ended survey, we were testing interest in participating in a future trial; therefore, we felt a subjective outcome was best suited for this study as there are many factors that may ultimately influence a decision to participate in a clinical trial, including availability, location, and incentives. Sociodemographic information, PEG score, pain location (back pain vs no back pain, knee pain vs no knee pain, and hip pain vs no hip pain), and the Media and Technology Usage Scale subscales were independent variables. We ran bivariate tests to determine whether independent variables were associated with patient interest in a hypothetical VR trial. For hypothesis testing in bivariate models, we set the double-sided α to .05. We created multivariate regression models using a stepwise approach. We identified variables a priori, including age, gender, and PEG score, to be included in the initial model regardless of significance. Additional covariates associated with the outcome in bivariate analyses at *P*<.20 were sequentially added to the model. Final model selection was guided by theoretical relevance, model fit, and statistical significance.

To summarize open-ended responses about participants’ interest in a VR trial, we coded responses using a thematic analysis framework. We reviewed codes and developed categories to reflect responses. After each response was categorized, we tabulated categories quantitatively. Where possible, we present illustrative text reflecting categories.

## Results

### Demographic and Survey Outcomes

#### Sociodemographic Variables

Of 693 participants contacted, 215 (31%) were successfully reached. Among those successfully reached, 124 (57.7%) declined, and 43 (20%) did not meet the inclusion criteria. The most common reason for exclusion in this study was only speaking Spanish (n=41, 15% of successful contacts). Of 172 potential participants, our final sample consisted of 48 (27.9%) individuals who met the inclusion criteria. The mean age of participants was 58.5 (SD 11.3) years. Of the participants, 32 (67%) were female and 16 (33%) were male. About half (n=23, 47.9%) of the participants had a high school education or less. About one-fifth (n=9, 19%) of participants were non-Hispanic White; 15 (31%) were Black; 18 (38%) were Hispanic; and 6 (13%) were Asian, Native Hawaiian or other Pacific Islander, or other. Please see Table 1 for detailed demographics.

**Table 1:** Survey participant characteristics (N=48).

Characteristic		Total cohort (n=48)	Interested (n=42)	Not interested (n=6)	*P* value	
Age (y), mean (SD; 95% CI)		58.5 (11.3; 55.2-61.9)	58.5 (11.1; 55.0-61.9)	58.7 (13.5; 44.5-72.8)	.97	
**Gender, n (%; 95% CI)**						.99
	Woman	32 (67; 52.5-78.9)	28 (67; 51.0-79.9)	4 (67; 29.6-90.9)		
**Education, n (%; 95% CI)**						.45
	High school or less	23 (48; 34.3-62.0)	21 (50; 35.3-64.7)	2 (33; 9.7-70.0)		
**Race or ethnicity, n (%; 95% CI)**						.99
	White, non-Hispanic	9 (19; 10.3-32.0)	8 (19; 9.8-33.6)	1 (17; 3.0-56.4)		
	Black, non-Hispanic	15 (31; 19.7-45.4)	13 (31; 19.0-46.3)	2 (33; 9.7-70.0)		
	Hispanic	18 (38; 24.9-52.0)	16 (38; 24.7-53.8)	2 (33; 9.7-70.0)		
	Other	6 (13; 5.6-25.6)	5 (12; 5.0-26.1)	1 (17; 3.0-56.4)		
**Pain severity, n (%; 95% CI)**						.89
	Moderate or higher	39 (81; 67.4-90.3)	34 (81; 66.7-90.1)	5 (83; 36.5-99.1)		
Pain intensity (PEG^a^ score), mean (SD; 95% CI)		6.31 (2.4; 5.61-7.01)	6.17 (2.5; 5.40-6.93)	7.28 (1.9; 5.31-9.25)	.30	
Texting use, mean (SD; 95% CI)^a^		5.7 (1.9; 5.2-6.2)	5.8 (1.8; 5.2-6.3)	5.1 (2.9; 2.1-8.1)	.45	
Smartphone use, mean (SD; 95% CI)^b^		4.2 (1.9; 3.6-4.7)	4.3 (1.9; 3.7-4.9)	3.3 (1.6; 1.6-5.0)	.21	
Email use, mean (SD; 95% CI)^b^		4.4 (2.2; 3.8-5.0)	4.6 (2.2; 4.0-5.3)	2.8 (2.1; 0.6-5.0)	.06	
Internet use, mean (SD; 95% CI)^b^		3.6 (3.0-4.2)	3.6 (3.0-4.3)	3.3 (1.3-5.3)	.75	
Facebook user^c^, n (%; 95% CI)		25 (2; 52.1; 37.9-66.0)	24 (2; 57.1; 41.0-71.9)	1 (2; 16.7; 3.0-56.4)	.06	

^a^PEG: Pain, Enjoyment of Life, and General Activity.

^b^Use is rated 1 to 10 on the Media and Technology Usage Scale, with higher values representing more use. The scale is as follows: 1=never, 2=once a month, 3=several times a month, 4=once a week, 5=several times a week, 6=once a day, 7=several times a day, 8=once an hour, 9=several times an hour, 10=all the time.

^c^Participants were asked if they have a Facebook account.

#### Pain Measures

The mean PEG score was 6.31 (SD 2.4), and 39 (81%) participants reported having pain that was at least moderate. Lower back pain was the most common pain site (n=30, 63%), followed by knee pain (n=17, 35%) and hip pain (n=14, 29%).

#### Media and Technology Usage Scale

The mean texting usage subscale score was 5.7 (SD 1.9), reflecting text message use between several times a week and once daily. The mean phone use was 5.6 (SD 1.8), again reflecting calling or receiving calls from others between several times a week and once daily. The mean smartphone usage subscale was 4.2 (SD 1.9), which reflects using smartphone functions about once per week. The mean email use was 4.4 (SD 2.2), and the mean internet use was 3.6 (SD 2.0), with both reflecting nondaily use of these technologies. Of the 48 participants, about half (n=25, 52%) reported using Facebook. Participants generally reported mixed attitudes toward technology; mean subscale scores on positive attitude scales with technology were 3.8 (SD 0.8), reflecting a stance approaching agreement; mean subscale scores on negative attitude scales were 3.1 (SD 1.0), reflecting a neutral stance; and mean subscale scores on anxiety or dependence on technology were 2.8 (SD 1.1), reflecting a neutral stance approaching disagreement that participants were anxious or dependent upon technology.

#### VR Questions

Of the 48 participants, about two-thirds (n=31, 65%) had never heard about VR, 41 (85%) had never tried a VR device, and 2 (4%) had heard about VR being used to treat pain. A small minority of participants (n=7, 15%) thought that VR could work for treating pain, with a majority (n=38, 79%) reporting being unsure. Despite this, about two-thirds (n=31, 65%) of participants were “very interested” in participating in a hypothetical study of at-home VR for CP, and an additional 11 (23%) were “somewhat interested.” As we had defined our primary outcome, interest in VR, as either “very interested” or “somewhat interested,” most participants (n=42, 88%) met the outcome. Only 6 (13%) participants reported not interested in participating in a hypothetical study of at-home VR for CP.

#### Factors Associated With Interest in Participating in a VR Trial

Relationships between (1) more frequent email use and (2) Facebook use and interest in participating in a VR clinical trial for pain approached statistical significance and were subsequently included in stepwise multivariate models (email subscale score among interested vs not interested participants: 4.6 vs 2.8; *P*=.06 and Facebook use among interested vs not interested participants: 24/42, 57% vs 1/6, 17%; *P*=.06). Age, gender, race, education, pain severity, pain location, smartphone use, and internet use were not predictive of being somewhat or very interested in participating in a VR clinical trial.

We examined predictors of interest across a series of multivariate models. Variables identified a priori included gender, age, and PEG score. These were added to a baseline model along with email use and Facebook use, which were identified through bivariate analyses. The initial model (model 1) was not statistically significant overall (*P*=.14). None of the individual predictors reached significance, though Facebook use approached significance (odds ratio [OR] 9.50, 95% CI 0.68-132.21; *P*=.09). After simplification (model 2: email use, PEG score, and Facebook use), the overall model became statistically significant (*P*=.04), and Facebook use remained a consistent predictor, although it did not reach statistical significance (OR 8.56, 95% CI 0.69-106.38; *P*=.10). In the final reduced model (model 3: PEG score and Facebook use), the model retained statistical significance (*P*=.04), and Facebook use emerged as a statistically significant predictor of VR interest (OR 11.37, 95% CI 1.04-124.81; *P*=.047).

### Open-Ended Qualitative Survey Responses: Reasons for Interest in VR

We collected 47 open-ended responses describing reasons for interest or lack of interest in participating in a VR clinical trial. We categorized responses into 7 separate categories. Two categories were related to interest: (1) interest in a new treatment modality and (2) desperation for pain relief. Four categories were related to lack of interest: (1) doubts about VR as a pain treatment, (2) fear of COVID-19, (3) scheduling and traveling concerns, and (4) lack of more information. Three coded responses could not be labeled as interest or lack of interest and instead were marked as “other.” The most common category was interest in a new treatment modality (27/47, 58%), followed by desperation for pain relief (9/47, 19%). The most common category representing lack of interest was doubts about VR as a pain treatment (3/47, 6%), followed by scheduling and traveling concerns (2/47, 4%). A sample quote reflecting a participant’s interest was as follows:

You never know what will work. There’s always advancements and new things that they discover outside of pills. I’m open to anything.

A sample quote reflecting a participant’s lack of interest was as follows:

My pain can only be helped by a doctor. No way virtual reality works for pain. If your boss is telling you that then they are lying.

## Discussion

### Principal Findings

In this telephone survey of 48 persons with CP, we found that, despite a lack of experience with and knowledge of VR, most participants were interested in a study of at-home VR for CP. In this racially and ethnically diverse sample, we found relatively infrequent use of smartphones, email, internet, and social media. Additionally, we found mixed evidence that technology use was predictive of interest in participating in a clinical trial for VR. In open-ended responses, reasons for interest tended to cite new treatment modalities and the high burden of CP. These results indicate that the desire for new and innovative technological interventions exists even among people with CP with relatively low technology use.

Our results did not find patient-level barriers to participating in technology-oriented clinical trials, including a lack of trust. Prior studies have indicated that mistrust of research institutions and investigators is a significant attitudinal barrier to research participation reported by African Americans [20]. Overall, non-Hispanic Black and Hispanic individuals exhibit much higher levels of medical mistrust than their White counterparts [21]. Nonetheless, our qualitative data did not suggest that mistrust was a major barrier for participants. Other patient-level systemic barriers, such as scheduling and transportation, were more prominent in open-ended responses. These barriers may restrict participation in clinical research, and any trial that seeks diverse participation should consider efforts to reduce systemic barriers in recruitment procedures.

By contrast, institutional barriers and researcher-level barriers can be a prominent systemic reason for exclusion in clinical trials. Institutional barriers can include inadequate or untimely reimbursement of participants, insufficient attention to research important to communities of racial and ethnic minority people, and challenges with institutional review boards and community partnerships [22]. Researcher-level barriers can include bias, among many other factors. A notable form of bias in pain research is the underrecognition of pain in minorities by physicians [23]. The belief that Black persons feel less pain than White persons remains prevalent among US physicians, leading to inadequate treatment recommendations and possibly lower referral to pain research [23,24]. Perceptions that individuals from certain racial and ethnic groups are challenging or not ideal study candidates also remain prevalent and lead to less diverse participation [25]. In this study, one important researcher-level barrier participants encountered was that we required English proficiency. Because we were conducting a cross-sectional survey of interest in participation in a VR trial, we elected to only include people who could participate in future VR studies. As of 2025, none of the current US Food and Drug Administration–approved devices for CP are available in Spanish.

The high level of interest in VR in this study implies that institutional and researcher-based systemic barriers could be more prominent for this population than patient-level barriers. Previous literature in other intervention modalities has been mixed on this topic. When given the opportunity to participate in clinical research, some studies show that racial and ethnic minority groups are as likely to participate as White populations, whereas others report the opposite [26-29]. One overarching conclusion of the literature to date is that opportunity to participate or interest in participating alone is often not enough, as actual participation still lags due to a myriad of factors. Ensuring that research opportunities are relevant and significant to populations of interest is absolutely critical, as is lowering systemic barriers to participation, such as by providing off-hour availability or transportation reimbursement.

Additionally, our results challenge the perception that a population characterized by low technology use has less interest in participating in clinical trials involving technology. We found mixed evidence that technology use was related to interest in VR. Though we found that Facebook use was associated with VR interest in multivariate models, other proxy measures for technology use, including internet use, were not similarly related. We theorize that, perhaps aside from technological familiarity, participants who maintained social ties on the internet may have a particular interest in technological solutions to their CP. For the entire sample, the high interest we observed may also reflect ongoing frustration with the treatment options provided by the health care system among patients with CP. In our open-ended responses, some participants indicated that they were desperate for pain relief and willing to try novel interventions. The extent to which participants feel more comfortable with technology-based research compared to traditional clinical research involving medications or other medical interventions, owing to historic mistrust and inequities of traditional research, is unclear and warrants further research.

Health care systems that adopt VR into routine pain management can increase patient interest and subsequent participation through increased exposure. For example, the Veterans Health Administration (VHA), the largest integrated health care system in the United States, is in the early stages of VR adoption for CP, with patients showing high levels of interest [30]. The VHA has established an Office of Healthcare Innovation and Learning and a specific VA Immersive Program, which has supported literature reviews, conferences, and innovative pilot programs in VR studies [31]. However, because of unique payment incentives, public and private hospital systems lag behind the VHA in technology adoption [32]. Nonetheless, there are new financial options to sustain VR care. Although VR for CP is not routinely covered by Medicare, VR devices can be covered if they are declared reasonable and necessary [33]. Reimbursement through Medicare may lead to increased adoption in private health care systems, such as ours at Montefiore, and affiliated public hospitals that serve diverse populations.

### Limitations

The primary limitation of this study is the lack of generalizability of our sample to the patient population. Our sample was small and hospital based, which may have introduced response bias. Some participants may have previously participated in clinical trials. Additionally, we were unable to reach many of our potential participants, and as such, we enrolled a relatively small proportion of patients we attempted to contact. Our telephone response rate is similar to recruitment rates in other studies we have conducted, but there may have been selection bias in the participants that responded to our phone call. However, the directionality of the bias is unclear; patients who answer telephone calls and use telephones more often may be less technologically savvy compared with those who communicate more with friends and relatives via SMS text message or email. By contrast, as observed in our previous studies and those of others withing our health care system and community [34], patients with economic challenges may not have consistent telephone access or consistent telephone numbers and may represent groups that are less technologically savvy. Additionally, we estimated engagement in clinical trial activities by asking about subjective interest, which may overestimate intent to participate because of response bias. We did not assess negative interactions with the health care system and how these may have affected interest in VR clinical trials. Nonetheless, to our knowledge, this is the first study to gauge the level of interest in a VR clinical trial among a predominantly Hispanic and Black patient population with CP.

### Conclusions

Patient populations of low SES and racial and ethnic minority groups are not sufficiently included in pain clinical trial recruitment. In this study, we found high interest in participating in a VR study for CP despite low technology use and lack of knowledge of VR, underscoring the importance of not underestimating patient willingness to engage with innovative treatments for CP. Further research is necessary to include diverse populations to ensure equity and effectiveness of VR, especially considering the high burden of CP among these populations. Addressing language accessibility and structural challenges, such as transportation and Medicare or Medicaid reimbursement, will be essential to ensure inclusive clinical trial recruitment and adoption of VR for CP.

## Abbreviations

CP: chronic pain
OR: odds ratio
PEG: Pain, Enjoyment of Life, and General Activity
SES: socioeconomic status
VHA: Veterans Health Administration
VR: virtual reality
